# Administration of a CXCL12 Analog in Endotoxemia Is Associated with Anti-Inflammatory, Anti-Oxidative and Cytoprotective Effects *In Vivo*

**DOI:** 10.1371/journal.pone.0138389

**Published:** 2015-09-16

**Authors:** Semjon Seemann, Amelie Lupp

**Affiliations:** Institute of Pharmacology and Toxicology, Jena University Hospital, Friedrich Schiller University Jena, Jena, Germany; National Institutes of Health, UNITED STATES

## Abstract

**Background:**

The chemokine receptor CXCR4 is a multifunctional receptor which is activated by its natural ligand C-X-C motif chemokine 12 (CXCL12). As CXCR4 is part of the lipopolysaccharide sensing complex and CXCL12 analogs are not well characterized in inflammation, we aimed to uncover the systemic effects of a CXCL12 analog in severe systemic inflammation and to evaluate its impact on endotoxin induced organ damages by using a sublethal LPS dose.

**Methods:**

The plasma stable CXCL12 analog CTCE-0214D was synthesized and administered subcutaneously shortly before LPS treatment. After 24 hours, mice were sacrificed and blood was obtained for TNF alpha, IFN gamma and blood glucose evaluation. Oxidative stress in the liver and spleen was assessed and liver biotransformation capacity was determined. Finally, CXCR4, CXCL12 and TLR4 expression patterns in liver, spleen and thymus tissue as well as the presence of different markers for apoptosis and oxidative stress were determined by means of immunohistochemistry.

**Results:**

CTCE-0214D distinctly reduced the LPS mediated effects on TNF alpha, IFN gamma, ALAT and blood glucose levels. It attenuated oxidative stress in the liver and spleen tissue and enhanced liver biotransformation capacity unambiguously. Furthermore, in all three organs investigated, CTCE-0214D diminished the LPS induced expression of CXCR4, CXCL12, TLR4, NF-κB, cleaved caspase-3 and gp91 phox, whereas heme oxygenase 1 expression and activity was induced above average. Additionally, TUNEL staining revealed anti-apoptotic effects of CTCE-0214D.

**Conclusions:**

In summary, CTCE-0214D displayed anti-inflammatory, anti-oxidative and cytoprotective features. It attenuated reactive oxygen species, induced heme oxygenase 1 activity and mitigated apoptosis. Thus, the CXCR4/CXCL12 axis seems to be a promising target in the treatment of acute systemic inflammation, especially when accompanied by a hepatic dysfunction and an excessive production of free radicals.

## Introduction

Administration of bacterial endotoxin represents a well-established animal model to investigate systemic inflammation and is frequently used to study the host’s innate immune response [1]. One of the main structures that play an important role in the innate immune system is represented by the toll-like receptors (TLR) necessary for the recognition of several pathogen-associated molecular patterns. Exposure of mammals to LPS leads, in particular, to an increased release of pro-inflammatory cytokines through TLR4-signaling. LPS hereby binds the lipopolysaccharide binding protein and interacts with a receptor complex formed by CD14 (cluster of differentiation 14), MD-2 (myeloid differentiation protein-2) and TLR4, which then results in TLR4 transduced signals that in turn lead to an increased expression of NF-κB, proteases and reactive oxygen and nitrogen species [2]. In septic and acute inflammatory diseases, widespread activation of TLR4 has been detected by different authors leading to the conclusion that TLR4 activation has to be limited [3]. It is known that other extracellular proteins—such as heat shock proteins (HSP 70 and HSP 90), CD55 and the chemokine receptor 4 (CXCR4) are able to influence TLR4 signaling, thus representing an interesting target to prevent TLR4 activation [4,5]. Concerning CXCR4, its importance has been demonstrated by showing that it is part of the "LPS-sensing apparatus" [6], suggesting that intervention with CXCR4 agonists or antagonists could result in reduced TLR4 signaling. Furthermore, intervention with a CXCR4 ligand could weaken LPS effects which are triggered by LPS binding to CXCR4 directly. Naturally, CXCR4 is activated by its ligand CXCL12, also known as stromal cell-derived factor 1 (SDF-1), which represents a multifunctional cytokine. CXCL12 is constitutively expressed by various cells and tissues, and exhibits chemo-attractive activity for monocytes, bone marrow neutrophils, and early stage B cell precursors [7,8]. It is also a highly efficient and potent chemoattractant for T cells, as well as a co-stimulator of their activation [9]. Moreover, CXCR4 deficient mice are not able to survive [10]. This impressively demonstrates the physiological importance of the CXCR4/CXCL12 axis. Recently, the diagnostic value has been shown in neonatal sepsis, where elevated CXCR4 and CXCL12 levels were accompanied by increased sepsis severity [11]. The involvement of the CXCR4/CXCL12 axis in autoimmune and inflammatory diseases is undisputed as treating rheumatoid arthritis, inflammatory bowel disease or lupus erythematodes with CXCR4-antagonists revealed beneficial outcomes [12–14]. As it turned out, CXCR4 and CXCL12 occurred above average in the affected tissue, so inhibition of the CXCR4/CXCL12 axis resulted in lower inflammatory responses—probably because less CXCR4 positive cells entered the tissue. In contrast, activation of the CXCR4 seemed to have positive effects on such systemic and acute diseases as sepsis or polytrauma and, there is evidence that in vivo treatment with CXCL12 analogs results in improved survival [15,16]. Nevertheless, the existing data concerning the role of the CXCR4/CXCL12 axis in inflammatory diseases remains contradictious and up to now, no further investigations were carried out to determine what exact effects a CXCL12 analog exerts in endotoxemia systemically and in the organs. We are the first to investigate the impact of a CXCL12 analog on a sublethal dose of endotoxin as we tested the plasma stable derivative of CXCL12, CTCE-0214D, and its influence on LPS induced organ damages. In contrast to others, we did not aim to investigate the impact of CTCE-0214D on the mortality rate as there are already data available which prove a beneficial influence of the CXCL12 analog CTCE-0214 in vivo both without and with co-treatment with other drugs [15,17,18]. Hence, we applied a sublethal dose of endotoxin, because we aimed to understand the effects mediated through the CXCL12 analog and to confirm the previously shown positive impact on septic shock. We focused on liver, spleen and thymus function to evaluate a possible beneficial impact of the treatment and to gain an idea concerning the underlying processes and effects. In addition, we were interested in the CXCR4/CXCL12 expression patterns and how these were changed during disease progression.

## Material and Methods

### CTCE-0214D

As CXCL12 is known to have a short half-life, we designed the plasma stable peptide CTCE-0214D in analogy to the peptide CTCE-0214 originally developed by Chemokine Therapeutics. CTCE-0214D was custom synthesized by Centic Biotec, Heidelberg, Germany. By using a four-glycine linker, the N-terminal region (residues 1–14) was connected to the C-terminal region (residues 55–66) of CXCL12, while (in contrast to CTCE-0214) the last amino acid (asparagine) was substituted by aspartate to avoid license problems. As previously shown, slight deviations in C terminal regions barely affect affinity to and activation of the CXCR4 [19,20]. The analogue is cyclized between the amino acid residues at the positions 20 and 24 by an isopeptide bond. To achieve plasma stability, the two cysteins were replaced by alanine and phenylalanine, respectively. The amino acid sequence of the peptide as one-letter code is as follows: KPVSLSYRAPFRFF-GGGG-LKWIQEYLEKALD.

### Animals and experimental procedure

The study was conducted under the licence of the Thuringian Animal Protection Committee (Reg-Nr.: 02-044/14). The principles of laboratory animal care and the German Law on the Protection of Animals as well as the Directive 2010/63/EU were followed. Male adult C57BL/6N mice (12-weeks-old, body weight 25–30 g; Charles River Laboratories, Sulzfeld, Germany) were used. The animals were housed in plastic cages under standardized conditions (light-dark cycle 12/12 h, temperature 22 ± 2°C, humidity 50 ± 10%, pellet diet Altromin 1316, water ad libitum). A total of 28 mice was randomly divided into four groups (n = 7 each): Control, LPS, CTCE-0214D and CTCE-0214D plus LPS. LPS (E. coli 0111:B4, Sigma Aldrich, Steinheim, Germany) was injected intraperitoneally (5 mg/kg body weight), whereas CTCE-0214D (20 mg/kg body weight) was administered in 0.9% saline shortly before endotoxemia onset subcutaneously. The most appropriate LPS dose was determined in a pilot study, in which we administered 1 mg/kg, 5 mg/kg or 10 mg/kg body weight LPS, respectively. As 5 mg/kg body weight LPS caused no mortality and the LPS effect was strong enough to detect possible beneficial effects after co-treatment with potential drugs, we chose this dosage for our further investigations. The dose of CTCE-0214D was selected based on previous publications, which investigated the CXCL12 analog CTCE-0214 in vivo [15, 21,22]. As we aimed a prolonged effect of the CXCL12 analog, we administered CTCE-0214D subcutaneously and chose a higher concentration than the minimum effective dose of 10 mg/kg body weight. The study design was chosen as the subcutaneous administration results in a delayed release and thereby in a prolonged effect, as the drug has to be initially absorbed, whereas the intraperitoneal LPS injection causes an immediate inflammatory response. 24 hours after LPS treatment, body temperature was measured and the mice’s condition was assessed by using the Clinical Severity Score (CSS) described previously [23]. Afterwards, the mice were sacrificed in isoflurane anesthesia and their liver, spleen and thymus were removed, weighed and either fixed in 10% buffered formaldehyde or snap-frozen in liquid nitrogen for biochemical analysis. Additionally, whole-blood was collected and blood sugar levels were determined using a commercially available blood glucose meter and respective test strips (BG star®, Sanofi-Aventis, Frankfurt, Germany). Subsequently, serum was obtained and used for ELISA measurements. To perform histological analysis, the formalin-fixed organ samples were embedded in paraffin blocks and cut into 4 μm thin sections (n = 7 for each treatment group).

### IFN-γ, TNF-α, aspartate aminotransferase (ASAT) and alanine aminotransferase (ALAT) assays

To determine the serum levels of interferon-γ, TNF-α, ASAT and ALAT, mouse IFN-γ ELISA kit (Pierce Biotechnology, Rockford, IL, USA), mouse TNF-alpha Quantikine ELISA kit (R&D Systems, MA, USA), EnzyChrom™ Aspartate Transaminase Assay Kit or EnzyChrom™ Alanine Transaminase Assay Kit (both BioAssay Systems, Hayward, CA, USA), respectively, were used according to the manufacturer’s instructions.

### Oxidative status in the tissues

The tissue content of glutathione in its reduced (GSH) and oxidized (GSSG) form was analyzed by homogenizing the samples with eleven volumes of 0.2 M sodium phosphate buffer (5 mM EDTA; pH 8.0) and four volumes of 25% metaphosphoric acid. After centrifugation (12000g, 4°C, 30 min) GSH content was measured in the supernatants using a colorimetric assay as previously described [24]. The GSSG concentration was assessed fluorometrically [25]. To determine the tissue content of lipid peroxides (LPO) as thiobarbituric acid reactive substances (TBARS), liver samples were homogenized with 19 volumes of ice-cold saline and analyzed fluorometrically [26]. Additionally, HO-1 (heme oxygenase 1) activities were measured in the liver 9000g supernatants (prepared as described under”biotransformation capacity“) using hemin as a substrate. The amount of bilirubin formed was determined photometrically and referred to the incubation time and to the protein content of the respective 9000g supernatants [27].

### Biotransformation capacity

To obtain 9000g supernatants, the livers were homogenized with 0.1 M sodium phosphate buffer (pH 7.4) (1:2 w/v) and subsequently centrifuged at 9000g for 20 minutes at 4°C. Activities of all biotransformation reactions were assessed in these 9000g supernatants and referred to the protein content of this fraction which was determined with a modified Biuret method [28]. For assessment of cytochrome P450 (CYP) enzyme activities, the following model reactions were performed: benzyloxyresurofin-O-debenzylation (BROD; [29]), ethoxycoumarin-O-deethylation (ECOD; [30]), ethoxyresorufin-O-deethylation (EROD; [31]), ethylmorphine-N-demethylation (EMND; [32]), methoxyresorufin-O-demethylation (MROD; [31]), p-nitrophenol-hydroxylation (PNPH; [33]), pentoxyresorufin-O-depentylation (PROD; [31]). Glutathione-S-transferase (GST) activities were determined by photometrically measuring the resulting dinitrobenzene-glutathione conjugate, GS-DNB [34].

### Histopathology and immunohistochemistry

Four-μm-sections were prepared from the paraffin blocks and floated onto positively charged slides. Immunostaining was performed by an indirect peroxidase labeling method as described previously [35]. Briefly, sections were dewaxed, microwaved in 10 mM citric acid (pH 6.0) for 16 min at 600 W and then incubated with the respective primary antibodies (Table 1) at 4°C overnight. Detection of the primary antibody was performed using either a biotinylated goat anti-rabbit IgG, rabbit anti-goat IgG or horse anti-mouse IgG, respectively, followed by an incubation with peroxidase-conjugated avidin (Vector ABC “Elite” kit, Vector, Burlingame, CA, USA). Binding of the primary antibody was visualized using 3-amino-9-ethylcarbazole (AEC) in acetate buffer (BioGenex, San Ramon, CA, USA). The sections were then rinsed, counterstained with Mayer's hematoxylin and mounted in Vectamount™ mounting medium (Vector Laboratories, Burlingame, CA, USA). Additionally, TUNEL (TdT-mediated dUTP-biotin nick end labeling) staining was performed by using the In Situ Cell Death Detection Kit, POD (Roche Diagnostics, Mannheim, Germany) according to manufacturer’s instructions. All immunohistochemical stainings were evaluated by two independent investigators. In the case of a discrepancy in the scoring between the two investigators a final decision was achieved by consensus. In spleen and thymus, the occurrence was assessed as follows: 0: negative; 1: seldom; 2: frequent; 3: diffuse. Additionally, the intensity of staining was evaluated as follows: 0: no staining; 1: mild; 2: moderate; 3: strong. In the livers, occurrence and staining intensity of Kupffer and pit cells were determined using the same rating. To detect the glycogen content in the livers, periodic-acid-Schiff staining (PAS; periodic acid, Schiff’s reagent: Sigma Aldrich, Steinheim, Germany) was performed by using standard protocols [36]. Identification of the specific cell types was based on their microscopic features along with the relative location of the cells in the respective tissues.

**Table 1:** Primary antibodies used for the immunohistochemical investigations.

Primary antibody	Type, Catalogue number	Manufacturer	Dilution	Host species
**CXCR4**	monoclonal, 3108–1	Epitomics	1:50	Rabbit
**CXCL12**	monoclonal, MAB350	R&D Systems	1:500	Mouse
**TLR4**	polyclonal, sc-12511	Santa Cruz Biotechnology	1:500	Goat
**NF-κB**	monoclonal, sc-8008	Santa Cruz Biotechnology	1:500	Mouse
**cleaved caspase-3**	monoclonal, 9661	Cell Signaling Technology	1:600	Rabbit
**gp91 phox**	polyclonal, sc-5827	Santa Cruz Biotechnology	1:500	Goat
**Heme oxygenase 1**	polyclonal, SPA-895	Biomol GmbH	1:5000	Rabbit

### Statistical Analysis

All statistical analyses and figures were computed with GraphPad Prism 6.0 software (GraphPad Software, La Jolla, CA, USA). All experiments were performed with seven animals per experimental group and statistical significance was determined by using the one-way analysis of variance (ANOVA) and the Tukey post hoc test. A p value of less than 0.05 (*) was considered as statistically significant; a p value of less than 0.01 (**) and a p value of less than 0.001 (***) are denoted separately. Data are given as mean ± standard error of the mean (SEM).

## Results

### Blood glucose, body temperature and general condition

**Fig 1:**
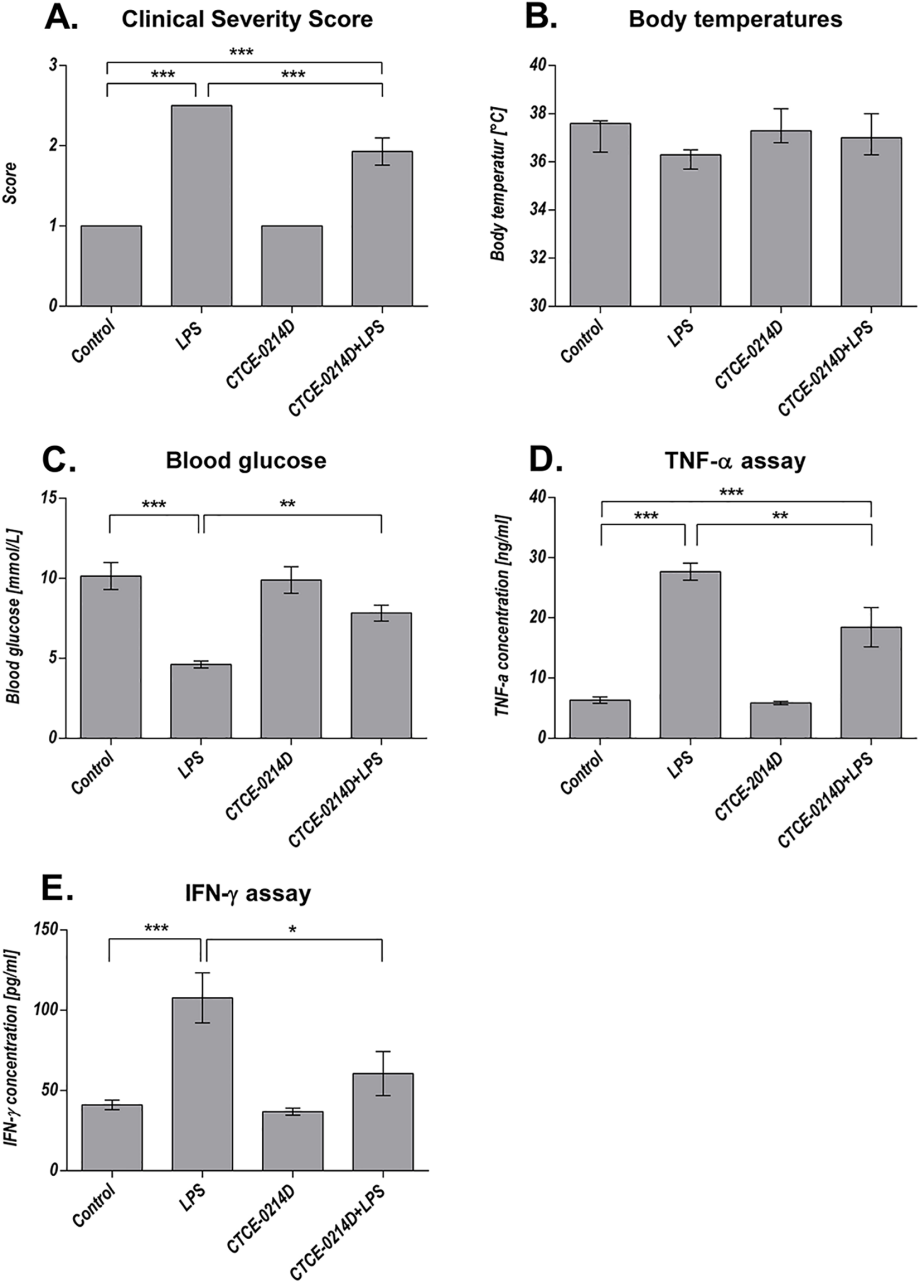
Clinical Severity Score, body temperatures, blood glucose, serum TNF-α and IFN-γ levels. 24 hours after LPS administration, the Clinical severity score was improved after CTCE-0214D plus LPS treatment as compared to mice which had received LPS only (A, 1.9±0.4 vs. 2.5±0, p≤0.001). In addition, endotoxic mice showed reduced body temperatures in comparison to the control (B, 36.2±0.2°C vs. 37.2±0.3°C, p = 0.09) and to the CTCE 0214D plus LPS group (37.1±0.3°C, vs. LPS p = 0.14). Moreover, a decrease of blood glucose levels (C) by about 50% when compared to the control group was seen (4.6±0.2 mmol/L vs. 10.1±0.9 mmol/L, p≤0.001). The CTCE-0214D plus LPS group showed a blood sugar level of 7.8±0.5 mmol/L, which is equivalent to an elevation by more than 65% when compared to endotoxin treatment only (p≤0.01). In addition, no significance was detectable in comparison to the control group. Serum TNF-α concentrations (D) were significantly reduced after co-administration of CTCE-0214D and LPS (27.7±1.4 ng/mL vs. 18.5±3.3 ng/mL, p≤0.01), while LPS caused an increase of more than 330% in comparison to the control group (6.6±0.5 ng/mL, p≤0.001). Similar results were achieved when determining serum IFN-γ levels (E). Compared to the control, the LPS challenge provoked an increase of approximately 160% (107.7±15.6 pg/mL vs. 41.1±3.0 pg/mL, p≤0.001), while CTCE0214D plus LPS co-administration did not result in significantly elevated serum INF-γ concentrations (60.5±13.8 pg/mL vs. 41.1±3.0 pg/mL, p = 0.58). Statistical significance (p≤0.05) was determined by using the one-way analysis of variance (ANOVA) and the Tukey post hoc test. Data are given as mean ± standard error of the mean (SEM), n = 7; *, p≤0.05; **, p≤0.01; ***, p≤0.001.

To examine the systemic influence of the CXCL12 analog, blood sugar levels, body temperatures and body and organ weights of all animals were determined. Additionally, the general condition of the mice was assessed by using the Clinical Severity Score. 24 hours after treatment, blood glucose levels of LPS challenged mice were significantly reduced by more than 50% when compared to control animals, whereas CTCE-0214D plus LPS treated mice showed only a slight, but non-significant reduction of the values by about 20% when compared to the control group. However, in comparison to the LPS group, concomitant administration of CTCE-0214D to LPS treatment caused an increase in the blood glucose levels by more than 60% (p≤0.01). Mice receiving CTCE-0214D alone showed no relevant difference in blood glucose levels to the control group (Fig 1).

After 24 hours, endotoxic mice displayed a reduced body temperature in comparison to the control animals (36.2±0.2°C vs. 37.2±0.3°C, p = 0.09) and to the CTCE 0214D plus LPS group (37.1±0.3°C, vs. LPS, p = 0.14). In accordance with these results, the CSS was improved after CTCE-0214D plus LPS treatment as compared to mice which had received LPS only (1.9±0.4 vs. 2.5±0, p≤0.001). No relevant influence of CTCE-0214D alone was detectable. In contrast to the effects seen on body temperature and on general condition, the CXCL12 analog had no impact on LPS induced body weight reduction (both treatment groups approximately -3.0g).

### TNF-α and IFN-γ serum levels

To investigate the impact of CTCE-0214D on endotoxemia, we measured serum cytokine levels at sacrifice. Compared to the control group, administration of LPS caused an elevation of serum TNF-α by about 330%, whereas CTCE-0214D was able to reduce the LPS-induced increase in serum TNF-α levels by more than 30%. Similar results were found when serum INF-γ concentrations were determined. In comparison to the control group, the LPS challenge provoked an increase by about 160%, while CTCE-0214D plus LPS co-administration resulted in not significantly elevated serum INF-γ concentrations. In comparison to the LPS treated animals, the CTCE-0214D plus LPS group showed almost two-fold lower serum IFN-γ levels (p = 0.02). In both ELISAs, no relevant influence of CTCE-0214D alone was detectable (Fig 1).

### Liver: Oxidative stress, biotransformation capacity, cytokines and apoptosis

**Fig 2:**
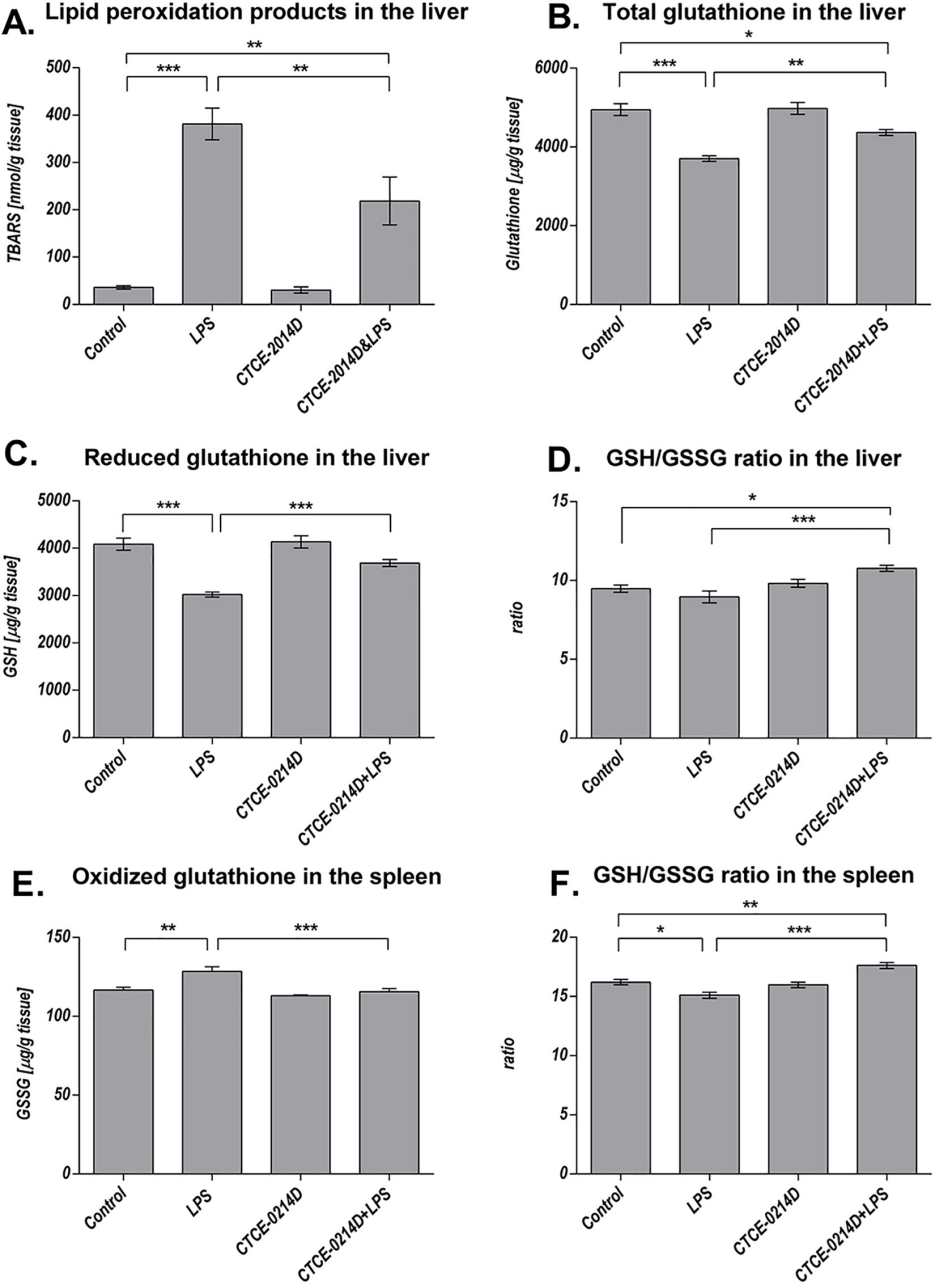
Oxidative stress in the liver and spleen. Tissue content of lipid peroxidation products as determined by thiobarbituric acid reactive substances (TBARS) in the livers of all animal groups 24 hours after treatment (A). CTCE-0214D reduced TBARS elevated due to endotoxemia to almost the half. As further parameters, total glutathione content (B), reduced glutathione levels (GSH, C) and the GSH/GSSG ratio were evaluated (D). In comparison to the control group, endotoxin caused a decrease in the total as well as reduced glutathione content and minimized the GSH/GSSG ratio, while co-administration of CTCE-0214D was able to mitigate all LPS effects significantly. Endotoxin caused a significantly reduced GSH/GSSG ratio in the spleen, whereas CTCE-0214D was able to increase the ratio which was attributable mainly to reduced GSSG levels. Statistical significance (p≤0.05) was determined by using the one-way analysis of variance (ANOVA) and the Tukey post hoc test. Data are given as mean ± standard error of the mean (SEM), n = 7; *, p≤0.05; **, p≤0.01; ***, p≤0.001.

**Fig 3:**
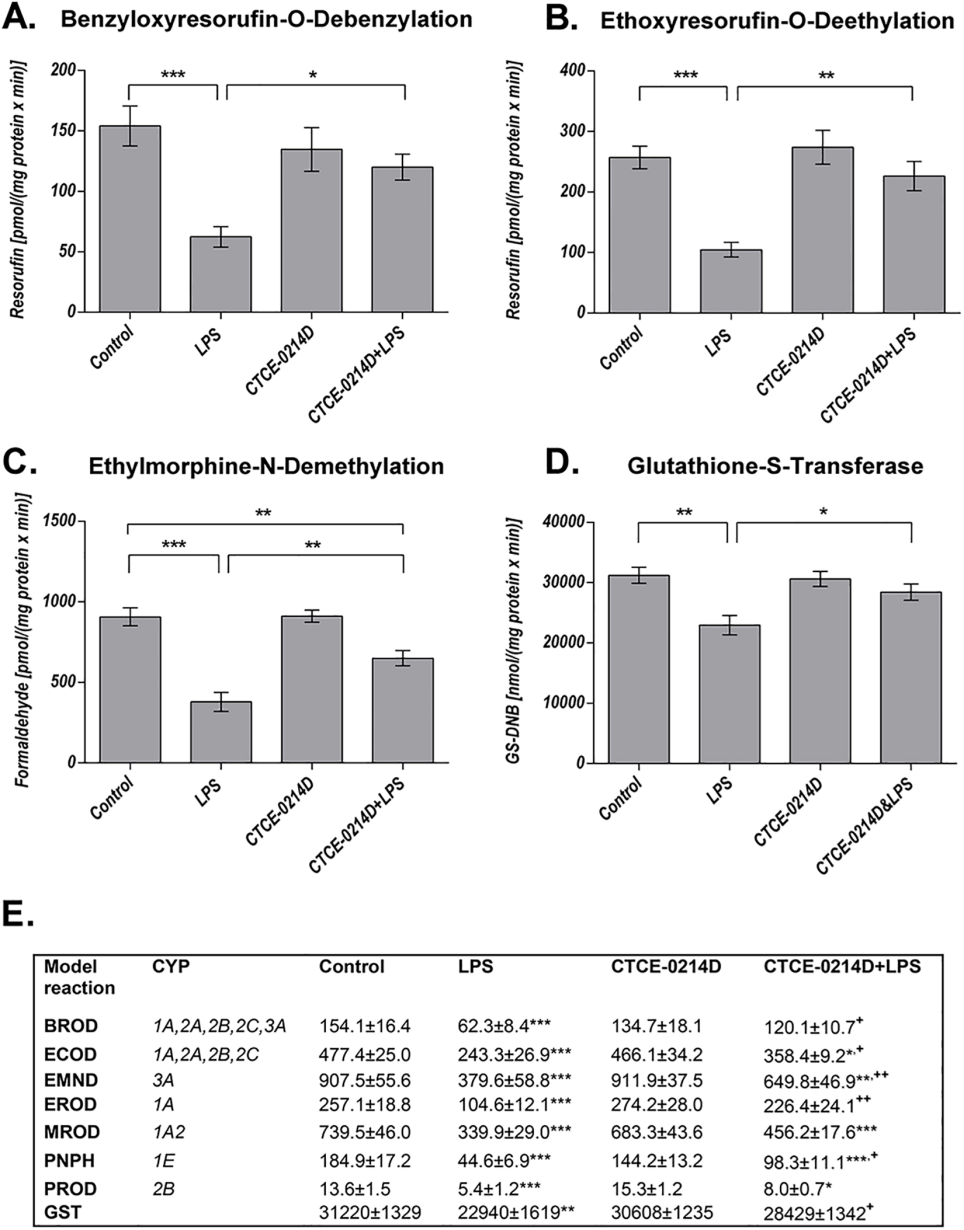
Biotransformation capacity. Benzyloxyresorufin-O-debenzylation [BROD] (A), ethoxyresorufin-O-deethylation [EROD] (B), ethylmorphine-N-demethylation [EMND] (C) and glutathione-S-transferase (D, as 1-chloro-2,4-dinitrobenzene conjugation) activities in 9000g supernatants are shown exemplarily. LPS impaired BROD activities to approximately 40% of the control values, whereas enzyme activities of CTCE-0214D plus LPS treated mice were elevated by more than 90% when compared to endotoxin administration alone. Moreover, no significant difference was detectable between CTCE-0214D plus LPS or saline treated mice. Furthermore, CTCE-0214D plus LPS treated mice revealed significantly elevated EROD activities when compared to LPS treatment, whereas no significance to the control group was detectable. Co-administration of CTCE-0214D and LPS ameliorated endotoxins effects on EMND activities (elevation by about 70%), the activity, however, still being significantly decreased when compared to control. Furthermore, increased glutathione-S-transferase activities were observed when comparing the CTCE-0214D plus LPS to the LPS group. Statistical significance (p≤0.05) was determined by using the one-way analysis of variance (ANOVA) and the Tukey post hoc test. Data are given as mean ± standard error of the mean (SEM), n = 7; *, p≤0.05; **, p≤0.01; ***, p≤0.001. In (E), the results of all performed model reactions are presented in pmol/(mg protein x min), except PNPH and GST, which are given in nmol/(mg protein x min). Statistical significance was determined and marked as mentioned, with the addition that significance to the LPS group was marked separately; ^+^, p≤0.05; ^++^, p≤0.01; ^+++^, p≤0.001 vs. LPS.

As the aminotransferases ASAT and ALAT are part of the diagnostic evaluation to determine liver health, we measured the serum levels of both enzymes (S2 Fig), however ALAT represents a more specific indicator of liver inflammation than ASAT. Endotoxin caused a massive increase in the serum concentration of both enzymes when compared to the control group (ASAT: 63.0±7.4 U/L vs. 98.3±9.0 U/L, p = 0.005; ALAT: 35.7±4.6 U/L vs. 85.7±16.5; p = 0.002). CTCE-0214D was able to decrease the enzyme activities throughout. The ASAT activities were reduced by about 15% when compared to the LPS group, whereas the ALAT activities showed a significant reduction of about 60% (p = 0.002). Remarkably, the ALAT activities of the control and of the CTCE-0214D plus LPS group are at the same level (35.7±4.6 U/L vs. 33.6±4.8 U/L, p = 0.98). To determine whether CTCE-0214D may attenuate LPS induced ROS production in liver tissue, the content of lipid peroxidation products (LPO) as well as the glutathione status was measured. Furthermore, we were interested in CYP and glutathione-S-transferase activities, which are known to be down regulated by endotoxin [37,38]. 24 hours after induction of endotoxemia, the livers of LPS treated mice had a more than 10 fold higher LPO concentration compared to control animals. Moreover, endotoxin caused a decrease in the total glutathione content to approximately 75% of the control level, whereas the GSH/GSSG ratio showed only a slight reduction. CTCE-0214D was able to diminish the LPS effects significantly. In doing this, the CXCL12 analog reduced the LPO content almost by half (vs. LPS p = 0.005) and was able to significantly increase the total glutathione content (vs. LPS p = 0.003)—corresponding to almost 90% of the control level. By increasing the reduced glutathione concentration by approximately 20% in regard to LPS (p≤0.001), the GSH/GSSG ratio was enhanced significantly (p≤0.001)—once again underlining CTCE-0214D's protective effects (Fig 2). In accordance with these results, the endotoxin-induced activity loss of several CYP families was attenuated. By determining CYP1A, 2A, 2B, 2C (ECOD) or CYP1A, 2A, 2B, 2C, 3A (BROD) activities, respectively, CTCE-0214D's protective effect was underlined impressively when compared to LPS treatment. Especially, when activities of specific CYP families were ascertained, LPS turned out to impair CYP1A activities to approximately 40% of the control values, whereas there was no significant difference in the values between CTCE-0214D plus LPS and saline treated mice. Similar results were observed when measuring activities of CYP1A2, CYP3A, CYP2B and CYP1E. In comparison to LPS alone, co-administration of the CXCL12-analog and endotoxin increased CYP activities by approximately 35%, 70%, 90% and 120%, yet being significantly decreased when compared to control levels. Fittingly, we witnessed increased glutathione-s-transferase activities of about 20% when comparing the CTCE-0214D plus LPS to the LPS group (Fig 3).

**Fig 4:**
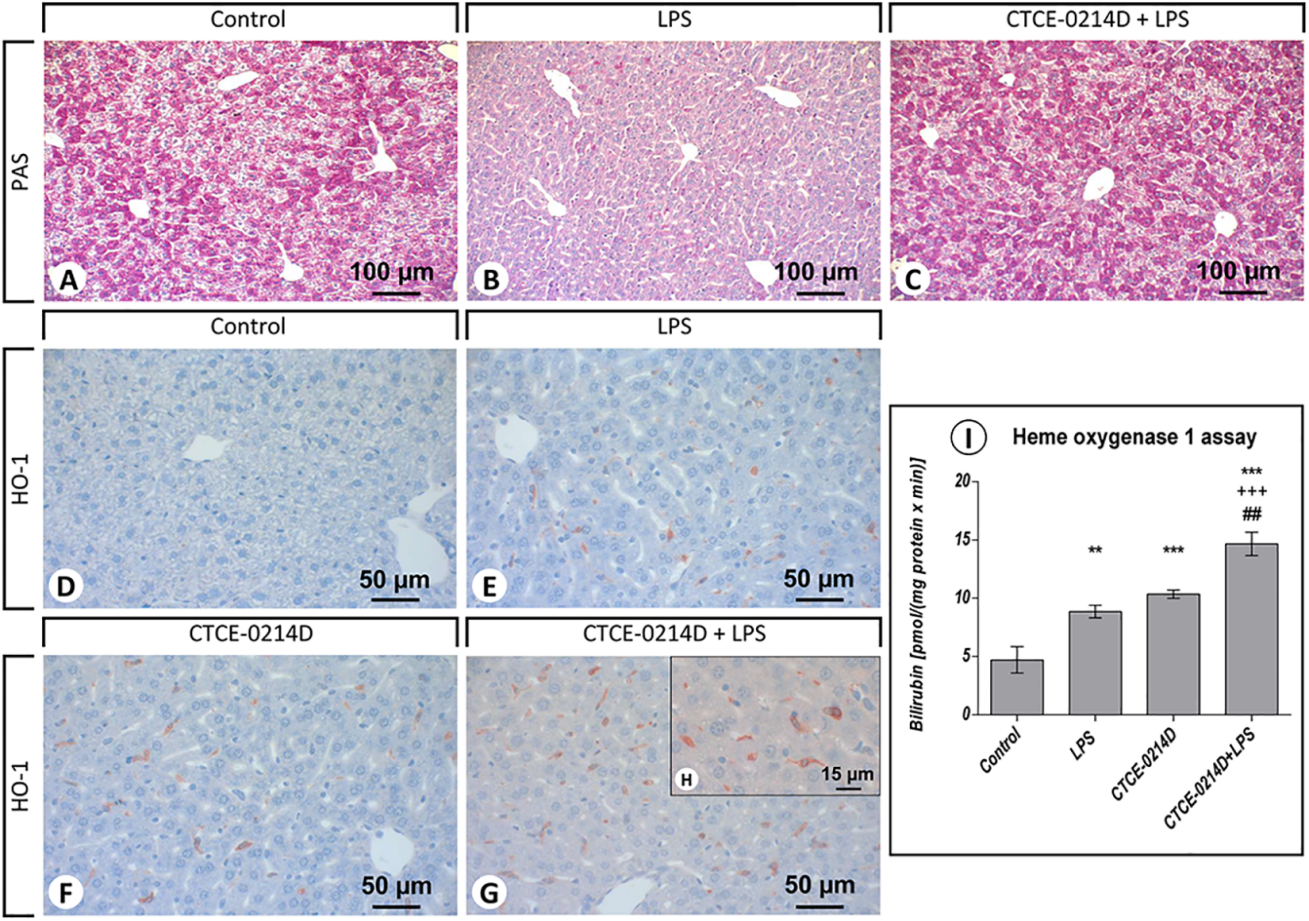
Periodic acid-Schiff (PAS) staining and immunohistochemical staining of heme oxygenase 1 in the livers. Representative photomicrographs from one of seven different tissue samples are shown (magnification: (A-C) 200x, (D-G) 400x, (H) 630x). For reasons of clarity, the PAS staining of the CTCE-0214D group is not shown as there were no differences to be observed when compared to the control. Exposure to LPS (B) evoked an impressive loss of glycogen content in the livers, whereas the livers of CTCE-0214D plus LPS treated mice (C) showed no difference to the control group (A) at all. LPS exposure (E) enhanced HO-1 occurrence in Kupffer and pit cells, whereas CTCE-0214D on its own (F) was able to induce HO-1 above average. Combined CTCE-0214D plus LPS administration (G) resulted in maximum HO-1 levels. The photomicrograph in (H) shows single Kupffer and pit cells, respectively, at a higher magnification (630x). To quantify the HO-1 activity in the liver, we performed an assay in the 9000g supernatants (I). LPS, CTCE-0214D and CTCE-0214D plus LPS administration increased the HO-1 activity by approximately 90%, 120% and 210%, respectively, when compared to the control group. The combined CTCE-0214D plus LPS treatment revealed the highest HO-1 activity levels, which were additionally significantly elevated in comparison to the values of the LPS or CTCE-0214D group, respectively. Statistical significance (p≤0.05) was determined by using the one-way analysis of variance (ANOVA) and the Tukey post hoc test. Data are given as mean ± standard error of the mean (SEM), n = 7; *, p≤0.05; **, p≤0.01; ***, p≤0.001 vs. control; ^+^, p≤0.05; ^++^, p≤0.01; ^+++^, p≤0.001 vs. LPS; #, p≤0.05; ##, p≤0.01; ###, p≤0.001 vs. CTCE-0214D.

To gain further information on the actions of CTCE-0214D in endotoxemia, we used Periodic acid-Schiff and immunohistochemial stainings. Exposure to LPS evoked an impressive loss of glycogen content in the livers, whereas the livers of CTCE-0214D plus LPS treated mice showed no difference to the control group at all (Fig 4A–4C). Moreover, many Kupffer and pit cells were perceivable after the LPS challenge, while the CXCL12 analog caused a reduction in these cells. Immunohistochemically, differences were noticeable in the amount and staining intensity of Kupffer and pit cells. Almost all hepatocytes were stained non-specifically. The administration of endotoxin led to an increased expression of CXCR4, NF-κB, cleaved caspase-3 and gp91 phox in Kupffer and pit cells (Table 2). Throughout, a staining of these target structures was seldom found in the livers of CTCE-0214D plus LPS treated mice. In fact, there was often no obvious difference recognizable when compared to the control group (Table 2). Suitably, CTCE-0214D's protective features were visible when observing HO-1 expression (Fig 4D–4H). While the antioxidant enzyme was evidently up-regulated after LPS exposure, CTCE-0214D plus LPS treatment caused the highest HO-1 expression in Kupffer and pit cells. In addition, it should be mentioned that the animals treated with CTCE-0214D only already showed a higher HO-1 expression throughout in comparison to the control group (Table 2 and Fig 4D–4H). In parallel to the immunohistochemical stainings also the HO-1 activity was measured in the liver 9000g supernatants. LPS, CTCE-0214D and CTCE-0214D plus LPS administration increased the HO-1 activity by approximately 90%, 120% and 210%, respectively, when compared to the control group. The combined CTCE-0214D plus LPS treatment revealed the highest HO-1 activity levels, which were additionally significantly elevated in comparison to the values of the LPS or CTCE-0214D group, respectively (Fig 4I).

**Table 2:** Immunohistochemical staining of CXCR4, CXCL12, TLR4, NF-κB, cleaved caspase-3, gp91 phox, heme oxygenase 1 and the TUNEL assay.

Staining	Treatment Group	Liver		Spleen				Thymus			
		*Kupffer and pit cells*		*Red pulp*		*White pulp*		*Cortex*		*Medulla*	
		*Occ*	*Int*	*Occ*	*Int*	*Occ*	*Int*	*Occ*	*Int*	*Occ*	*Int*
**CXCR4**	*Control*	1	1	1	1	1	1	2	1	2	1
	*LPS*	2	2	1	1	3	3	3	2	1	2
	*CTCE-0214D*	1	1	1	1	1	1	2	1	2	1
	*CTCE-0214D+LPS*	1	1	1	1	2	1	2	1	2	2
**CXCL12**	*Control*	2	2	1	2	2	2	1	2	2	2
	*LPS*	2	2	3	2	3	3	2	3	1	1
	*CTCE-0214D*	2	2	1	2	2	2	1	2	2	1
	*CTCE-0214D+LPS*	2	2	2	2	2	2	2	3	2	2
**TLR4**	*Control*	1	1	1	1	1	1	1	1	1	1
	*LPS*	2	2	2	1	3	3	2	1	1	1
	*CTCE-0214D*	1	1	1	1	1	1	1	1	1	1
	*CTCE-0214D+LPS*	1	1	1	1	2	1	1	1	1	1
**NF-κB**	*Control*	1	1	2	2	3	1	3	1	3	1
	*LPS*	2	2	2	2	3	3	3	3	3	1
	*CTCE-0214D*	1	1	2	2	3	1	3	1	3	1
	*CTCE-0214D+LPS*	1	1	1	2	3	1	3	1	3	1
**cleaved**	*Control*	0	0	1	2	1	2	1	2	1	3
**caspase-3**	*LPS*	2	2	1	2	3	3	3	3	1	3
	*CTCE-0214D*	0	0	1	2	1	2	1	2	1	3
	*CTCE-0214D+LPS*	1	1	1	2	1	2	1	2	1	3
**gp91 phox**	*Control*	1	1	1	1	0	0	1	1	1	1
	*LPS*	2	2	2	1	2	1	3	2	2	1
	*CTCE-0214D*	1	1	1	1	0	0	1	1	1	1
	*CTCE-0214D+LPS*	1	2	1	1	1	1	2	2	2	1
**Heme**	*Control*	0	0	1	1	0	0	1	1	1	1
**oxygenase 1**	*LPS*	2	2	2	1	2	2	1	1	1	1
	*CTCE-0214D*	2	2	2	2	0	0	1	1	1	1
	*CTCE-0214D+LPS*	3	3	3	2	2	2	1	1	1	1
**TUNEL**	*Control*	0	0	0	0	1	2	1	1	1	3
	*LPS*	2	2	1	2	3	3	3	3	1	3
	*CTCE-0214D*	0	0	0	0	1	2	1	1	1	3
	*CTCE-0214D+LPS*	1	1	0	0	1	2	1	1	1	3

Presented are the results in the tissues of liver, spleen and thymus of all treatment groups (each n = 7). Occurrence (Occ) was assessed as following: 0: negative; 1: seldom; 2: frequent; 3: diffuse. Additionally, the intensity of staining was evaluated as follows: 0: no staining; 1: mild; 2: moderate; 3: strong.

### Spleen: Oxidative stress, CXCR4/CXCL12 axis, TLR4, apoptosis and HO-1

To determine the influence of the CXCL12 analog on the oxidative status, we measured the glutathione level in splenic tissue. While the total glutathione content was almost similar throughout, the GSH/GSSG ratio of CTCE-0214D plus LPS treated mice was enhanced when compared to endotoxic ones, an effect, which was mainly due to significantly reduced GSSG concentrations (Fig 2).

**Fig 5:**
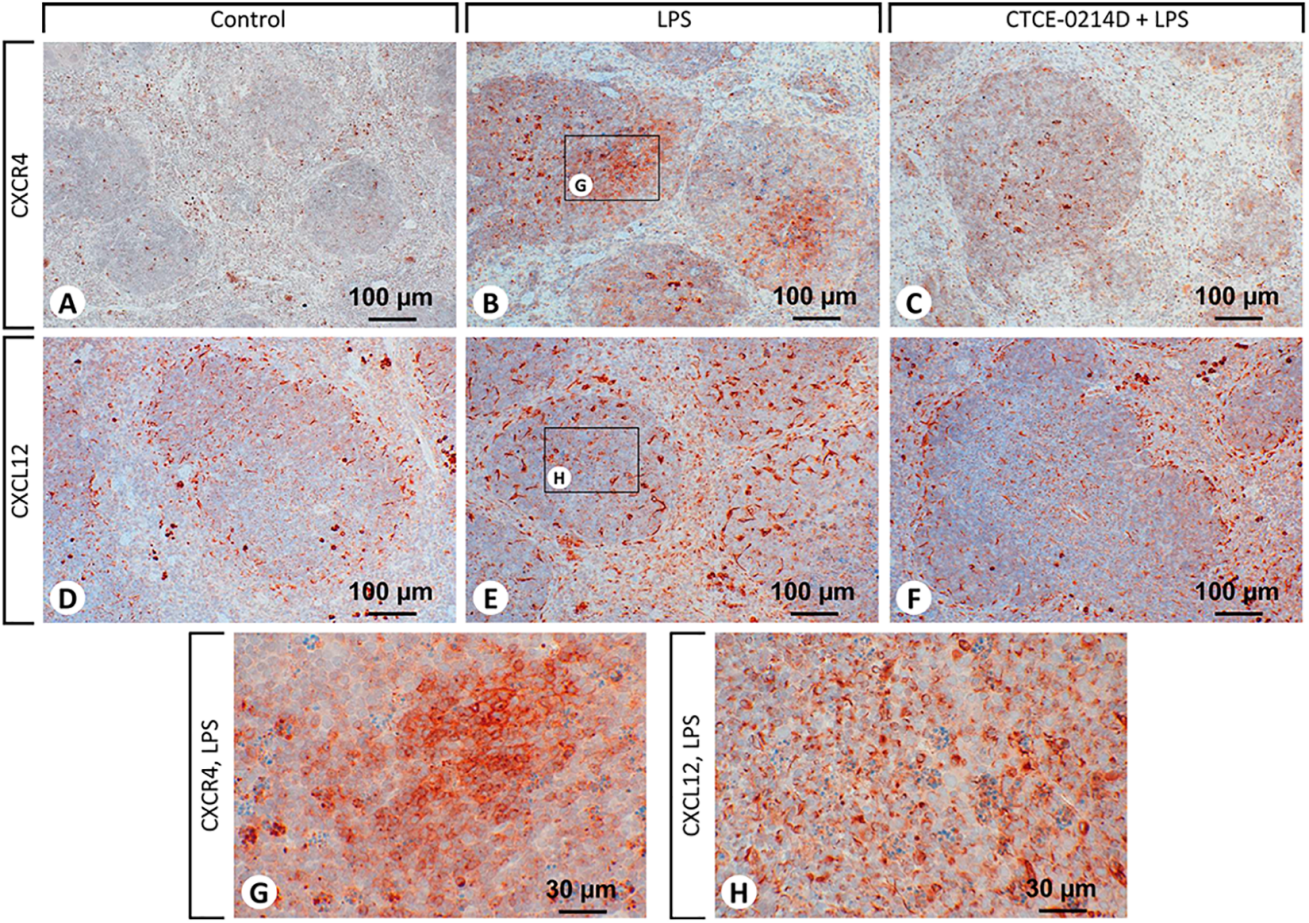
Immunohistochemical staining of CXCR4 and CXCL12 in the spleens. Representative photomicrographs from one of seven different tissue samples are shown (magnification: 200x). For reasons of clarity, the CTCE-0214D group is not shown as there were no differences to be observed when compared to the control. After saline treatment (A), CXCR4 appeared on macrophages and on lymphocytes in the red and in the white pulp, wherein in lymphoid follicles its presence was predominantly limited to lymphocytes in the marginal zone. Several cells in the red pulp are stained non-specifically (clearly distinguishable from the specific staining because of a granular appearance of the staining). LPS challenge (B) caused various CXCR4 positive cells to appear in the white pulp. The additional administration of CTCE-0214D diminished the amount of CXCR4 positive cells in the tissue to a minimum (C). CXCL12 staining in the spleen of saline treated animals (D) revealed that particularly splenocytes in the white and in the red pulp as well as plasma cells in the red pulp produced CXCL12. LPS treatment caused an increased CXCL12 expression in plasma cells, splenocytes and endothelial vascular cells in the red pulp, whereas in the white pulp predominantly splenocytes and tingible body macrophages became positive. Co-administration of CTCE-0214D to the endotoxin (F) evidently reduced the appearance of CXCL12. In the photomicrographs of (G) and (H) the tingible body macrophages (CXCL12 positive) and the engulfed lymphocytes in their cell bodies (CXCR4 positive) are depicted at a higher magnification (630x).

Immunohistochemistry revealed a correlation concerning the occurrence of CXCR4 and its ligand CXCL12 (Fig 5). After saline treatment, CXCR4 appeared on macrophages and lymphocytes in the red and white pulp, wherein in lymphoid follicles its presence was principally limited to lymphocytes in the marginal zone. After LPS treatment, CXCR4 positive cells were noticed only rarely in the red pulp, but the stronger the course of the disease, the more of these CXCR4 positive cells were found to be located in the white pulp. Accordingly, the CTCE-0214D plus LPS group displayed fewer CXCR4 positive cells in the splenic tissue (Table 2 and Fig 5A–5C and 5G). When focusing on CXCL12 staining in the spleens of saline treated animals, we ascertained particularly splenocytes in the white and red pulp and plasma cells in the red pulp to produce CXCL12 (Fig 5D). Obviously, endotoxin caused a widespread occurrence of CXCL12 all over the spleen (Fig 5E and 5H). While a staining of plasma cells, splenocytes and endothelial vascular cells appeared in the red pulp, an immunoreactivity of splenocytes and tingible body macrophages occurred predominantly in the white pulp (Table 2 and Fig 5D–5F and 5H). The more CXCL12 positive cells appeared in the spleen, the more CXCR4 positive cells were detectable. Especially the tingible body macrophages (CXCL12 positive) and the engulfed lymphocytes in their cell bodies (CXCR4 positive) support this hypothesis.

**Fig 6:**
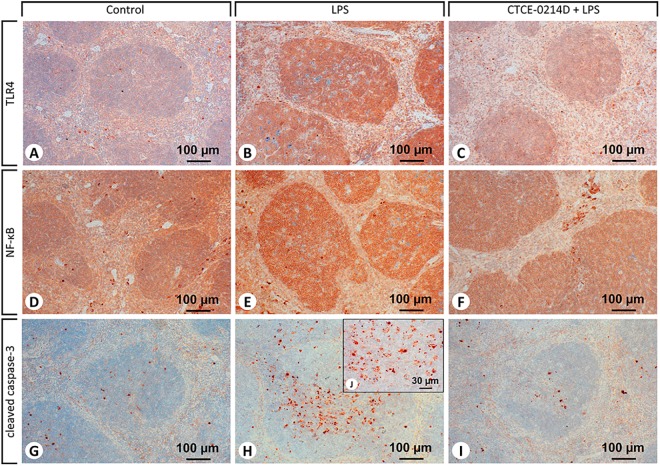
Immunohistochemical staining of TLR4, NF-κB and cleaved caspase-3 in the spleens. Representative photomicrographs from one of seven different tissue samples are shown (magnification: (A-I) 200x, (J) 630x). For reasons of clarity, the CTCE-0214D group is not shown as there were no differences to be observed when compared to the control. TLR4 occurred primarily in the white pulp, whereas cells of the red pulp displayed almost no membrane-bound staining. Especially after LPS treatment (B), spleno- and lymphocytes were identified to show an intensive TLR4 expression. Co-administration of CTCE-0214D to LPS clearly decreased the staining intensity of NF-κB, as lympho—and splenocytes in lymphoid follicles of the white pulp displayed diminished NF-κB levels (F). Nevertheless, the nuclear factor occurred ubiquitously in the spleens of all groups. Besides a few spleno- and lymphocytes, especially tingible body macrophages showed advanced cleaved caspase-3 activity and were found unambiguously more often in LPS-challenged mice (H,J). Co-administration of CTCE-0214D plus LPS reduced the appearance of cleaved caspase-3 to a minimum (I).

**Fig 7:**
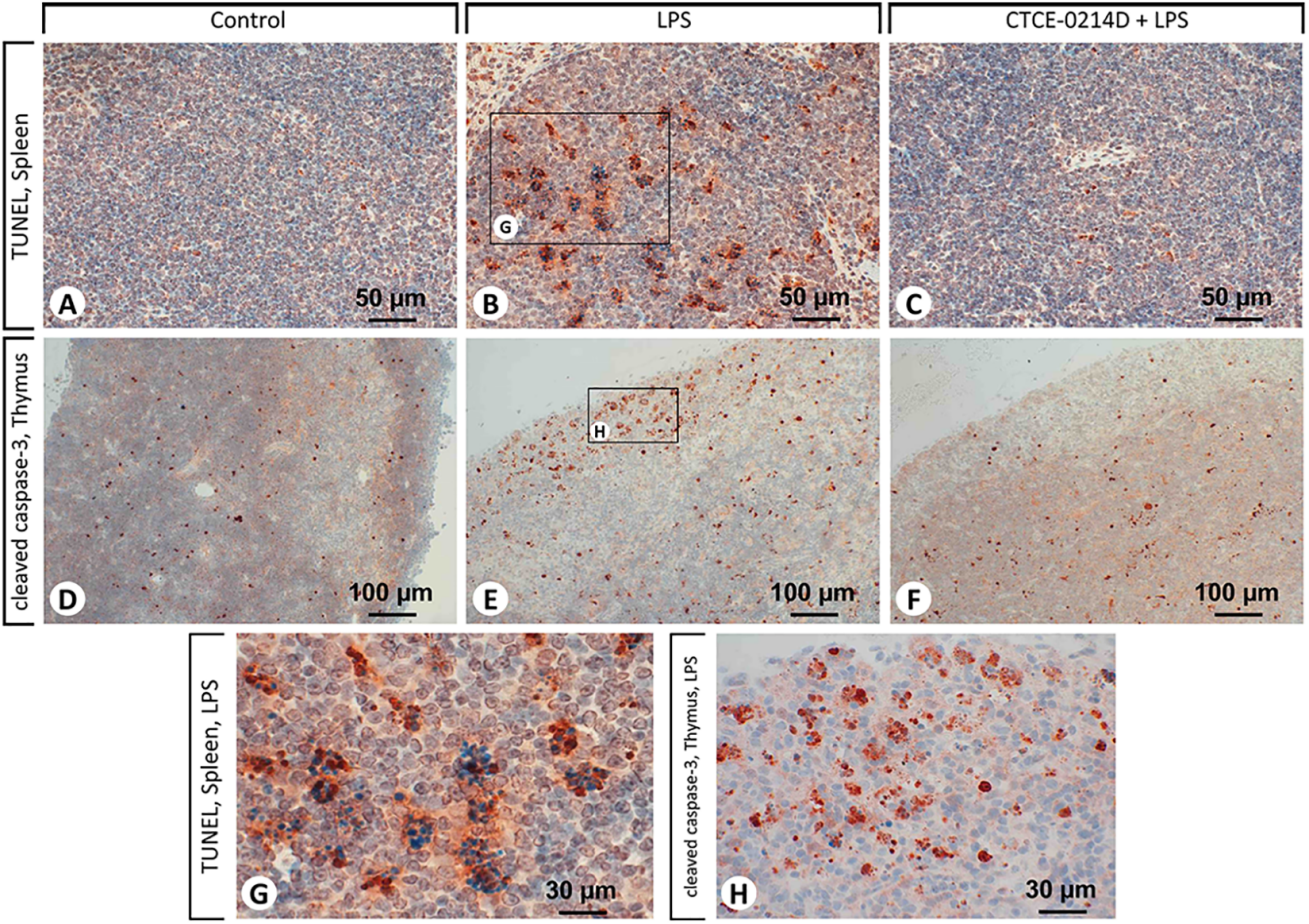
TUNEL assay in the spleens and immunohistochemical staining of the cleaved caspase-3 in the thymi. Representative photomicrographs from one of seven different tissue samples are shown (magnification: (A-C) 400x, (D-F) 200x, (G,H) 630x). For reasons of clarity, the CTCE-0214D group is not shown as there were no differences to be observed when compared to the control group. Whereas endotoxin induced cell death in both the white and red pulp (B), CTCE-0214D was able to significantly diminish the amount of cells undergoing apoptosis (C), which is particularly evident in the white pulp. In the thymi of the LPS group, endotoxin caused a massive appearance of cleaved caspase-3 in the cortex (E), whereas the medulla did not significantly differ from the other treatment groups. CTCE-0214D decreased the cleaved caspase-3 expression in the cortical regions to control level and caused a raise in the amount of lymphocytes (F), especially when compared to the LPS group. The magnifications in (G) and (H) reveal the tingible body macrophages in the white pulp to be the main locus of apoptotic cell death.

Continuing, TLR4 distribution in splenic tissue was confined primarily to the white pulp, whereas cells of the red pulp displayed almost no membrane-bound staining (Fig 6A–6C). Especially splenocytes and lymphocytes of septic spleens were identified to show an intensive TLR4 expression (Fig 6A–6C). Furthermore, when CTCE-0214D was administered in endotoxemia, lympho- and splenocytes in lymphoid follicles of the white pulp were found to exhibit decreased NF-κB activities (Fig 6E). In contrast to LPS and CTCE-0214D plus LPS administration, saline treated mice revealed a ubiquitous expression of the nuclear factor—particularly when compared to the red pulp (Table 2 and Fig 6D–6F). Examination of the spleens from septic mice revealed cells in the white pulp to express cleaved caspase-3 above average (Fig 6H and 6J). Besides a few spleno- and lymphocytes, especially tingible body macrophages showed an increased cleaved caspase-3 expression and were found unambiguously more often in LPS-challenged mice in contrast to other treatment groups. In the white as well as in the red pulp, CTCE-0214D reduced the appearance of cleaved caspase-3 to a minimum (Table 2 and Fig 6G–6J). The TUNEL assay confirmed the results, as spleens of CTCE-0214D plus LPS treated mice revealed a significantly reduced amount of cells undergoing apoptosis (Fig 7). In contrast, endotoxin induced a massive cell death, especially in the white pulp (Fig 7A–7C and 7G). Furthermore, the red pulp of LPS challenged mice clearly showed a higher perfusion which was detectable by the more frequent appearance of erythrocytes.

Also, macrophages and splenocytes in the white pulp of endotoxic mice were observed to express gp91 phox above average, whereas macrophages and neutrophil granulocytes in the red pulp showed similar expression levels in all treatment groups. In addition to its effects on the glutathione content, the beneficial features of CTCE-0214D were further underlined by the fact that LPS-induced gp91 phox activities in macrophages were ameliorated (Table 2). Suitably, the results of the HO-1 staining further underline the fact that administration of a CXCL12 analog is associated with anti-oxidative effects during inflammation. Similar to the staining results obtained in the liver, HO-1 expression was the highest after co-administration of CTCE-0214D plus LPS (Table 2). Especially macrophages in the red pulp and tingible body macrophages in the white pulp exhibited high enzyme activities. CTCE-0214D itself was able to induce HO-1 in macrophages and in granulocytes in the red pulp, while LPS treatment consistently caused the lowest expression of HO-1 in the red pulp. In the white pulp of LPS treated mice, HO-1 occurred predominantly on tingible body macrophages to a similar extent as after CTCE-0214D plus LPS co-treatment (Table 2).

### Thymus: CXCR4/CXCL12 axis, NF-κB and apoptosis

With respect to the CXCR4/CXCL12 axis, thymus immunohistochemistry revealed the same results as were seen in the spleen (Table 2). While tingible body macrophages expressed large amounts of CXCL12, CXCR4 occurred predominantly on the engulfed lymphocytes inside or in those located in close proximity. Furthermore, we recognized thymocytes in the medulla of the thymus as well as macrophages and endothelial cells in the cortex to be the major CXCL12 producers (not shown).

Moreover, almost every cell in the cortex of the thymus of LPS treated mice exhibited a high expression of NF-κB. In contrast to the other treatment groups, after LPS administration the medulla was additionally characterized by fewer lympho- and thymocytes, so consequently, a minimized NF-κB occurrence was observed. In addition, we were able to detect an atrophy of the cortical regions. The thymus of mice treated with CTCE-0214D plus LPS, however, also revealed an atrophy in cortex, but the medulla appeared to be enlarged as a result of more lympho- and thymocytes present in this region. Furthermore, co-administration of CTCE-0214D and LPS attenuated the NF-κB expression in cortical regions to control-like levels (not shown).

By using immunohistochemistry, we were able to perceive the influence of CTCE-0214D on the thymus, since a substantial raise in the amount of lymphocytes and a decrease in cleaved caspase-3 expression in the tissue was seen when compared to the LPS group. 24 hours after treatment, cleaved caspase-3 showed a remarkably high expression in the cortical regions of the thymi of septic mice, whereas the presence of this marker in the medulla did not differ from the other treatment groups. Very similar to the spleen, many tingible body macrophages were also recognized in the thymus tissue after LPS treatment, the cells engulfed being the main locus of apoptosis simultaneously. CTCE-0214D decreased the cleaved caspase-3 expression in the cortex to control level, while its presence in the medulla was not affected substantially (Table 2 and Fig 7D–7F and 7H). In accordance with these results, the TUNEL assay revealed the thymic cortex of LPS treated mice to be the main locus of apoptosis. We detected endotoxin to cause a massive cell death there, whereas CTCE-0214D was able to significantly mitigate apoptosis (Table 2).

## Discussion and Conclusions

It has been previously shown that by blocking the CXCR4/CXCL12 axis with synthetic antagonists during sepsis, the release of neutrophils from the bone marrow was prevented. This was accompanied by a 5 fold increase in peritoneal bacteria forming units in comparison to CLP (cecal ligation and puncture) treatment. Consequently, the mortality increased by about 40% [39]. Accordingly, when treating zebrafish with AMD 3100, a specific inhibitor of CXCR4, LPS treatment associated mortality was increased [40]. This finding is in good agreement with our results, as we were also able to observe a worsening of endotoxemia symptoms when additionally treating mice with AMD3100. In this case, significantly increased levels of serum TNF-α as well as lipid peroxidation products in the livers were seen, when compared to the control or LPS group, respectively. In addition, a significant loss of body weight was observed which was accompanied by reduced protein, total glutathione and CYP activity levels in the livers of co-treated mice (S1 Fig). On the other hand, the administration of exogenous CXCL12 was shown to induce a clear reduction in parasitemia after malarial infection in mice [41]. As CXCL12 is known to be highly expressed in the bone marrow of healthy animals to host CXCR4 positive cells, there is some evidence that the gradient is inverted in infectious diseases [39,17]. Our results support this hypothesis as we were able to show a higher content of CXCL12 producing cells in spleens of LPS challenged mice. Moreover, a larger amount of CXCR4 positive cells was detectable and the more CXCL12 appeared on tingible body macrophages the more CXCR4 positive cells were noticeable. Probably, the tingible body macrophages attract dysfunctional lymphocytes to remove them as a part of the host immune response to the endotoxin challenge. CTCE-0214D reduced the amount of tingible body macrophages in spleen and thymus indicating that the removal of pathogens was evidently more successful at the time of sacrifice. Consequently, exogenous CXCL12 administration appears to be a possibility to support the organism to mobilize functional CXCR4 positive cells to the local sites of infection, which is essential for survival. Our findings are in accordance with the recently described superiority of the combined treatment with CTCE-0214 and imipenem, in which neutrophil recruitment to the site of infection and bacterial clearance were improved in mice [17].

Additionally, we were able to detect an evidently reduced TLR4 appearance in spleens of mice treated with CTCE-0214D plus LPS. In the acute phase, endotoxic mice show a high expression of TLR4 which is known to be a physiological answer to a pathogen challenge and is predominantly caused by endotoxin and IFN-γ [42,43]. Consequently, LPS is able to activate receptor signaling which is accompanied by the enhanced activation of transcription factors like NF-κB. Considered along with the obviously diminished appearance of NF-κB in the liver, spleen and thymus, CTCE-0214D seems to distinctly attenuate TLR4 signaling. In addition, as LPS has been shown previously to directly activate CXCR4 [6], CTCE-0214D could antagonize LPS binding. Suitably, CXCL12 has been shown to suppress NF-κB activation in response to LPS stimulation in transfected HEK cells [44]. This is in accordance with the observed reduction of serum TNF-α levels, which may be a consequence of receptor antagonism or inhibition of NF-κB activation. This hypothesis is further supported by the decreased NF-κB and TNF-α production after administering ubiquitin in lethal endotoxemia, while ubiquitin was recently identified to be a CXCR4 agonist [45]. NF-κB plays a pivotal role in host immune response and functions as a key regulator by controlling cell proliferation, differentiation, apoptosis and immune and stress responses. Especially cyclooxygenase-2 (COX-2) is a well-known product of NF-κB activation. Produced by COX-2 induction, prostaglandins were formerly shown to stimulate glycogenolysis in the liver. Considered along with the enhanced metabolic activities in inflammation, which require many resources, the administration of endotoxin results in hypoglycemia [46,47]. Correspondingly, in the present investigation histological assessment revealed the livers of endotoxic mice to exhibit almost no glycogen reserves, while CTCE-0214D plus LPS treatment inhibited endotoxin effects completely. Suitably, activation of CXCR4 significantly enhanced blood glucose levels. Along with body temperature and general condition, blood glucose levels are of diagnostic value in identifying septic diseases [48], once again emphasizing CTCE-0214D's beneficial influence as the CXCL12 analog improved all parameters. Moreover, massive NF-κB activity results in the excessive production of pro-inflammatory cytokines such as TNF-α and IL-6. In particular, both are thought to play pivotal roles in endotoxemia by decreasing the biotransformation capacity in the liver to a minimum [37,49]. We determined CTCE-0214D to attenuate serum TNF-α levels, which is in good accordance to previous investigations concerning CTCE-0214 [15]. As a result, CTCE-0214D evidently improved phase 1 (CYP) and phase 2 (GST) enzyme activities. By doing so, CTCE-0214D probably improved the ability to detoxify LPS-induced secondary products such as reactive oxygen species. In addition, while septic patients are frequently treated with several drugs such as multiple antibiotics, the patients' biotransformation capacity is of relevant importance to detoxify and eliminate the resulting metabolites. As CTCE-0214D significantly recovered the biotransformation capacity, it could improve the patients' overall outcome as it may contribute to reduce the side effects caused by the multiple medications. Additionally, the more CYP families are active, the more proteins are protected from degradation [50]. Especially, reduced glutathione is of massive importance because of its diverse functions including detoxification, modulation of cell proliferation and antioxidant defense [51]. GSH has also been implicated in the modulation of cell death and is essential for cell survival as the GSH-depleted γ-glutamylcysteine synthetase knock-out mice died of massive apoptotic cell death [52,53]. As we were able to ascertain a beneficial glutathione status in both liver and spleen, this is probably one reason why CTCE-0214D exerts anti apoptotic effects. Furthermore, CXCL12 has been shown to inhibit cortical neuron apoptosis by increasing the ratio of Bcl-2/Bax after traumatic brain injury [54] and, also, pancreatic β cell survival was promoted by the activation of the prosurvival kinase Akt after CXCL12 administration [55]. Considered along with the reduced serum TNF-α and ROS levels in liver and spleen, CTCE-0214D probably provides its anti-apoptotic features in several distinct ways which may explain the massive reduction of apoptotic cells and the decreased expression of the cleaved caspase-3 witnessed in all organs.

Especially in the liver, endotoxin caused massive oxidative stress, which can be an important mediator of damage to cell structures, including lipids and membranes, proteins, and DNA [56]. CTCE-0214D, however, diminished LPS effects evidently which, in turn, is probably a result of several processes taking place simultaneously. Of key relevance is the attenuated occurrence of NF-kB, probably caused by attenuated TLR4 signaling. During inflammatory processes, the expression of phagocytic NADPH oxidase (gp91 phox) is dependent on, and induced by, NF-κB. Upon stimulation by growth factors and cytokines, the oxidase generates superoxide and other ROS by using NADPH [57]. Under normal circumstances, a mild activity of the NADPH oxidase is essential to eliminate bacteria and fungi. Endotoxin, however, was capable of amplifying the NADPH oxidase occurrence in the liver, spleen and thymus, while organs of mice treated with CTCE-0214D exhibited a moderate expression. Another potential reason for the observed reduction of ROS levels is probably due to the decreased serum TNF-α and IFN-γ levels as the cytokines have already been reported several times to generate ROS via mitochondria and NADPH oxidase [58,59]. Furthermore, in addition to NF-κB, both cytokines are able to heavily upregulate the inducible nitric oxide synthase (iNOS). Consequently, CTCE-0214D seems likely to attenuate iNOS´ ability to produce nitric oxide (NO). Besides producing cell toxic peroxynitrite, NO may contribute to hypotension, cardiodepression and vascular hyporeactivity in septic shock [60].

Finally, we demonstrated that CTCE-0214D is able to increase HO-1 activity in the liver as well as its expression in the liver and spleen. Once again, CTCE-0214D underlines its protective features as targeted overexpression of HO-1 has been shown to have beneficial effects in various experimental animal models of inflammation, a finding supported by the fact that HO-1 deficient mice are more susceptible to polymicrobial sepsis [61]. Moreover, previous investigations confirmed HO-1 to clearly attenuate TNF-α, ROS and NADPH oxidase levels [62,63] which is in good agreement with our results.

In summary, in the present investigation we were able to demonstrate that the CXCL12 analog CTCE-0214D displayed anti-inflammatory, anti-oxidative and cytoprotective features and thus was able to attenuate LPS-induced effects distinctly throughout. In contrast, blocking the CXCR4 with AMD3100 revealed to be disadvantageous and worsened the disease. As we are the first to reveal exact effects of CXCR4 activation, our results suggest the CXCR4/CXCL12 axis to be a promising target not only for the treatment of endotoxemia but also for the management of other acute inflammatory diseases. Especially when accompanied by an impaired liver function and an excessive production of free radicals, CXCL12 analogs represent a new treatment option to prevent and mitigate the disease progression.

## Supporting Information

S1 Fig Blockade of the CXCR4/CXCL12 axis with AMD3100 in endotoxemia. Male adult C57BL/6N mice (12-weeks-old, body weight 25–30 g; Charles River Laboratories, Sulzfeld, Germany) were used. The animals were housed in plastic cages under standardized conditions (light-dark cycle 12/12 h, temperature 22 ± 2°C, humidity 50 ± 10%, pellet diet Altromin 1316, water ad libitum). A total of 28 mice was randomly divided into four groups (n = 7 each): Control, LPS, AMD3100 and AMD3100 plus LPS. LPS (E. coli 0111:B4, Sigma Aldrich, Steinheim, Germany) was injected intraperitoneally (5mg/kg body weight), whereas AMD3100 (5 mg/kg body weight) was administered in PBS shortly after endotoxemia onset intraperitoneally. 24 hours after LPS treatment, mice were weighed and sacrificed in isoflurane anesthesia. Blood serum and livers were obtained and used for biochemical analysis. After combined treatment with AMD3100 plus LPS, significantly increased levels of serum TNF-α as well as lipid peroxidation products in the livers of mice when compared to the control or LPS group, respectively, were observed. In addition, a significant loss of body weight was observed which was accompanied by reduced protein, total glutathione and CYP activity levels in the livers of the co-treated mice. Statistical significance (p≤0.05) was determined by using the one-way analysis of variance (ANOVA) and the Tukey post hoc test. Data are given as mean ± standard error of the mean (SEM), n = 7; *, p≤0.05; **, p≤0.01; ***, p≤0.001 vs. control; ^+^, p≤0.05; ^++^, p≤0.01; ^+++^, p≤0.001 vs. LPS. These results clearly indicate that a blockade of the CXCR4/CXCL12 axis in endotoxemia is disadvantageous and worsens the disease. Undoubtedly, the chemokine receptor CXCR4 plays an important role in endotoxemia, and hence its activation becomes a promising treatment option not only for handling endotoxemia but also for other acute inflammatory diseases, especially when accompanied by an impaired liver function. The study was conducted under the licence of the Thuringian Animal Protection Committee. The principles of laboratory animal care and the German Law on the Protection of Animals as well as the Directive 2010/63/EU were followed.
(PDF)

S2 Fig Aspartate aminotransferase (ASAT) and alanine aminotransferase (ALAT) activities in the serum. LPS caused a massive increase in the serum concentration of both enzymes when compared to the control group (ASAT: 63.0±7.4 U/L vs. 98.3±9.0 U/L, p = 0.005; ALAT: 35.7±4.6 U/L vs. 85.7±16.5 U/L; p = 0.002). However, CTCE-0214D was able to decrease the enzyme activities throughout. The ASAT activities (A) were reduced by about 15% when compared to the LPS group (p = 0.5), whereas the ALAT activities (B) showed a significant reduction by about 60% (p = 0.002). Remarkably, the ALAT activities of the control and of the CTCE-0214D plus LPS group are at the same level (35.7±4.6 U/L vs. 33.6±4.8 U/L, p = 0.98). As ALAT represents a more specific indicator of liver inflammation than ASAT, these findings underline the protective effects of CTCE-0214D on the livers impressively.
(PDF)
