# Supplementary material for: Administration of a CXCL12 Analog in Endotoxemia Is Associated with Anti-Inflammatory, Anti-Oxidative and Cytoprotective Effects In Vivo
Source: PLoS One. 2015 Sep 16;10(9):e0138389. doi: 10.1371/journal.pone.0138389 (PMC4574197; doi:10.1371/journal.pone.0138389)
Supplement: S1 Fig — Male adult C57BL/6N mice (12-weeks-old, body weight 25–30 g; Charles River Laboratories, Sulzfeld, Germany) were used. The animals were housed in plastic cages under standardized conditions (light-dark cycle 12/12 h, temperature 22 ± 2°C, humidity 50 ± 10%, pellet diet Altromin 1316, water ad libitum). A total of 28 mice was randomly divided into four groups (n = 7 each): Control, LPS, AMD3100 and AMD3100 plus LPS. LPS (E. coli 0111:B4, Sigma Aldrich, Steinheim, Germany) was injected intraperitoneally (5mg/kg body weight), whereas AMD3100 (5 mg/kg body weight) was administered in PBS shortly after endotoxemia onset intraperitoneally. 24 hours after LPS treatment, mice were weighed and sacrificed in isoflurane anesthesia. Blood serum and livers were obtained and used for biochemical analysis. After combined treatment with AMD3100 plus LPS, significantly increased levels of serum TNF-α as well as lipid peroxidation products in the livers of mice when compared to the control or LPS group, respectively, were observed. In addition, a significant loss of body weight was observed which was accompanied by reduced protein, total glutathione and CYP activity levels in the livers of the co-treated mice. Statistical significance (p≤0.05) was determined by using the one-way analysis of variance (ANOVA) and the Tukey post hoc test. Data are given as mean ± standard error of the mean (SEM), n = 7; *, p≤0.05; **, p≤0.01; ***, p≤0.001 vs. control; +, p≤0.05; ++, p≤0.01; +++, p≤0.001 vs. LPS. These results clearly indicate that a blockade of the CXCR4/CXCL12 axis in endotoxemia is disadvantageous and worsens the disease. Undoubtedly, the chemokine receptor CXCR4 plays an important role in endotoxemia, and hence its activation becomes a promising treatment option not only for handling endotoxemia but also for other acute inflammatory diseases, especially when accompanied by an impaired liver function. The study was conducted under the licence of the Thuringian Animal Protection Commit [file pone.0138389.s001.pdf]

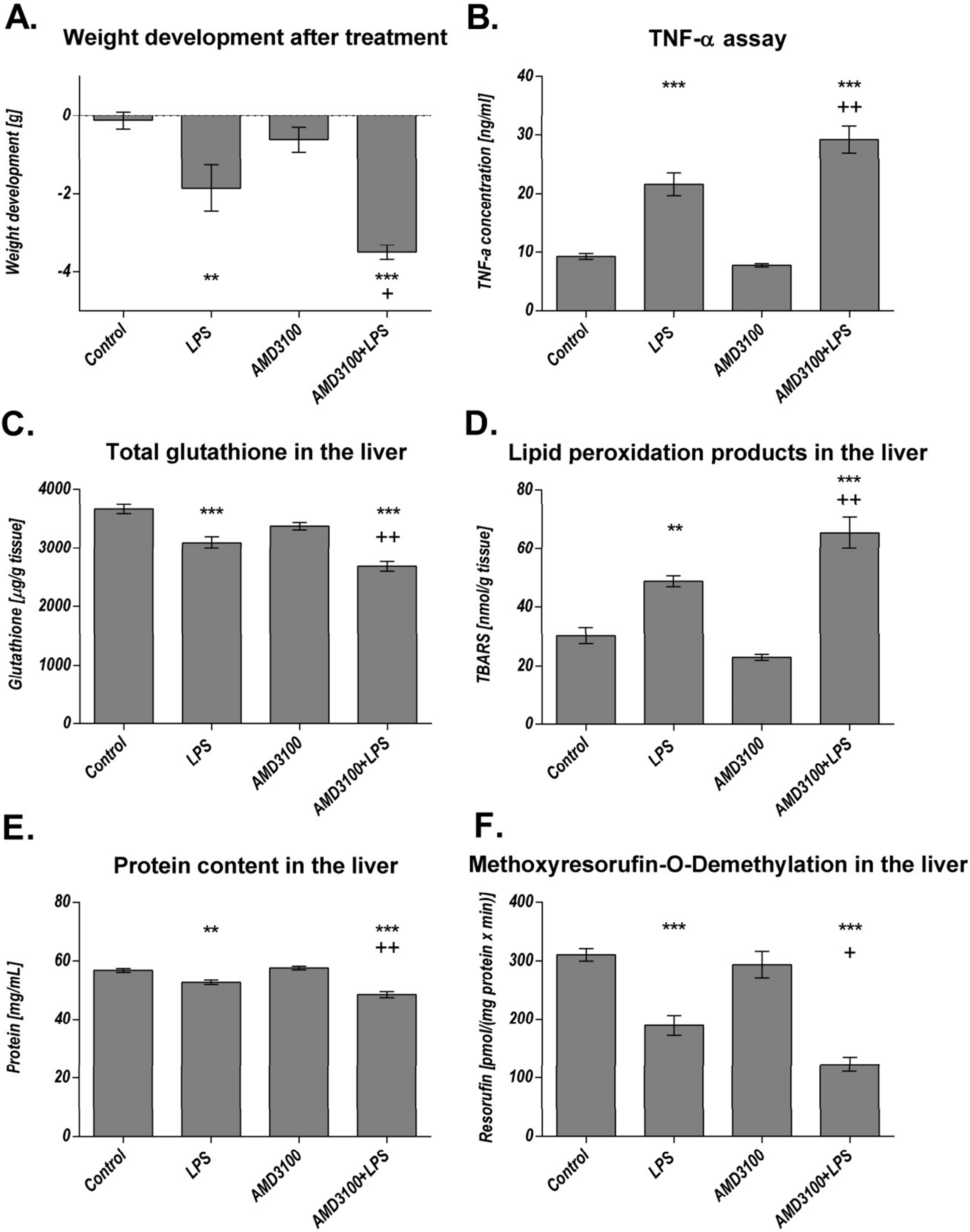
A.
Weight development after treatment
Weight development [g]
0
-2
-4
**
***
+
Control
LPS
AMD3100
AMD3100+LPS
B.
TNF-α assay
TNF-a concentration [ng/ml]
40
30
20
10
0
***
***
++
Control
LPS
AMD3100
AMD3100+LPS
C.
Total glutathione in the liver
Glutathione [μg/g tissue]
4000
3000
2000
1000
0
***
***
++
Control
LPS
AMD3100
AMD3100+LPS
D.
Lipid peroxidation products in the liver
TBARS [nmol/g tissue]
80
60
40
20
0
**
***
++
Control
LPS
AMD3100
AMD3100+LPS
E.
Protein content in the liver
Protein [mg/mL]
80
60
40
20
0
**
***
++
Control
LPS
AMD3100
AMD3100+LPS
F.
Methoxyresorufin-O-Demethylation in the liver
Resorufin [pmol/(mg protein x min)]
400
300
200
100
0
***
***
+
Control
LPS
AMD3100
AMD3100+LPS
