# Supplementary figures and images for: Administration of a CXCL12 Analog in Endotoxemia Is Associated with Anti-Inflammatory, Anti-Oxidative and Cytoprotective Effects In Vivo
Source: PLoS One. 2015 Sep 16;10(9):e0138389. doi: 10.1371/journal.pone.0138389 (PMC4574197; doi:10.1371/journal.pone.0138389)

## A. ASAT

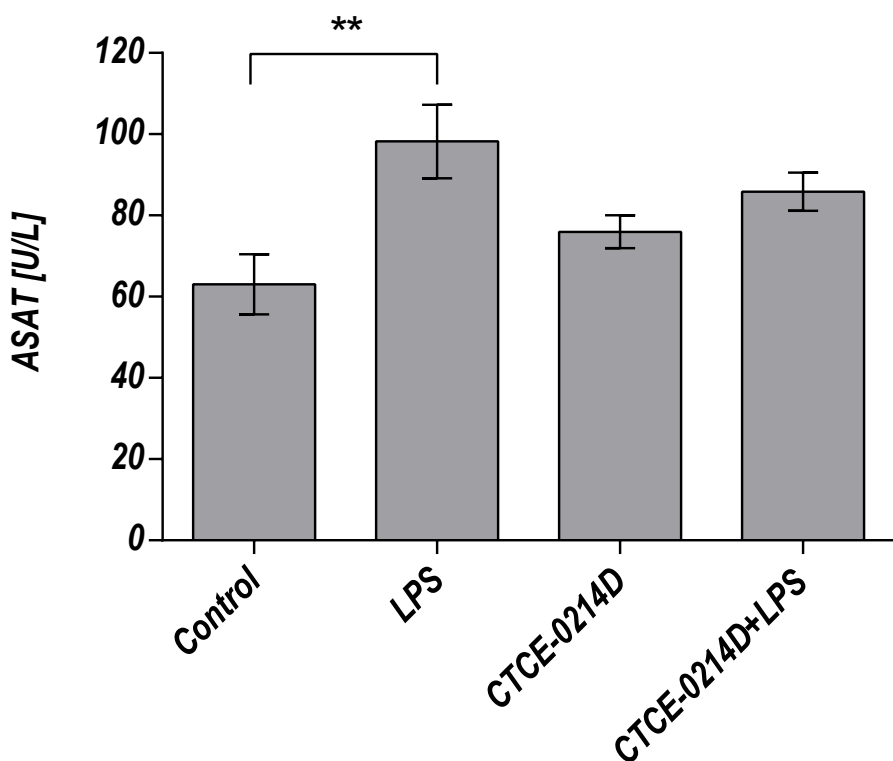

## B. ALAT

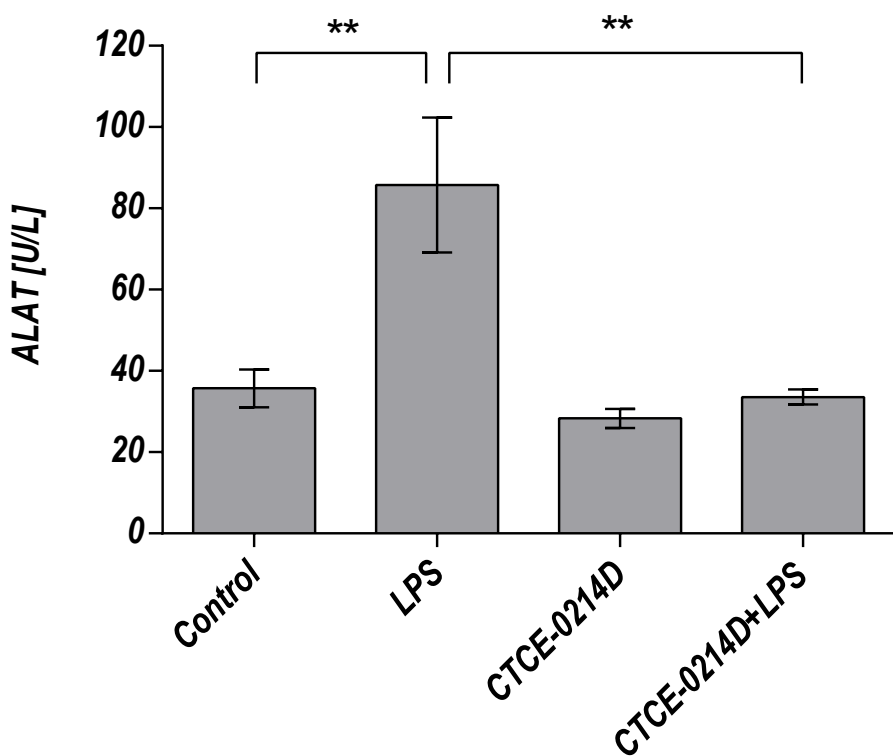

Supplement: S2 Fig — LPS caused a massive increase in the serum concentration of both enzymes when compared to the control group (ASAT: 63.0±7.4 U/L vs. 98.3±9.0 U/L, p = 0.005; ALAT: 35.7±4.6 U/L vs. 85.7±16.5 U/L; p = 0.002). However, CTCE-0214D was able to decrease the enzyme activities throughout. The ASAT activities (A) were reduced by about 15% when compared to the LPS group (p = 0.5), whereas the ALAT activities (B) showed a significant reduction by about 60% (p = 0.002). Remarkably, the ALAT activities of the control and of the CTCE-0214D plus LPS group are at the same level (35.7±4.6 U/L vs. 33.6±4.8 U/L, p = 0.98). As ALAT represents a more specific indicator of liver inflammation than ASAT, these findings underline the protective effects of CTCE-0214D on the livers impressively. (PDF) [file pone.0138389.s002.pdf]
